# Supplementary material for: Seed germination and vegetative and in vitro propagation of Hieracium lucidum subsp. lucidum (Asteraceae), a critically endangered endemic taxon of the Sicilian flora
Source: PeerJ. 2024 Feb 9;12:e16839. doi: 10.7717/peerj.16839 (PMC10860557; doi:10.7717/peerj.16839)

**Binary Logistic Regression: Germination versus Temperature; Solution**

**Method**

| Link function | Logit |
| --- | --- |
| Categorical predictor coding | (1; 0) |
| Rows used | 16 |

**Response Information**

| **Variable** | **Value** | **Count** | **Event Name** |
| --- | --- | --- | --- |
| Germination | Event | 267 | Germination |
|  | Non-event | 53 |  |
| Trials | Total | 320 |  |

**Regression Equation**

| P(Germination) | | | = | exp(Y')/(1 + exp(Y')) |  |
| --- | --- | --- | --- | --- | --- |
| Y' | = | 2.585 + 0.0 Temperature_15°C - 0.447 Temperature_20°C - 1.127 Temperature_25°C - 1.544 Temperature_30/15°C + 0.0 Solution_GA3 - 0.142 Solution_pure water | | | |

**Coefficients**

| **Term** | **Coef** | **SE Coef** | **VIF** |
| --- | --- | --- | --- |
| Constant | 2.585 | 0.455 |  |
| Temperature |  |  |  |
| 20°C | -0.447 | 0.553 | 1.98 |
| 25°C | -1.127 | 0.508 | 2.30 |
| 30/15°C | -1.544 | 0.493 | 2.41 |
| Solution |  |  |  |
| pure water | -0.142 | 0.308 | 1.00 |

**Odds Ratios for Categorical Predictors**

| **Level A** | **Level B** | **Odds Ratio** | **95% CI** |
| --- | --- | --- | --- |
| Temperature |  |  |  |
| 20°C | 15°C | 0.6395 | (0.2165; 1.8894) |
| 25°C | 15°C | 0.3241 | (0.1197; 0.8779) |
| 30/15°C | 15°C | 0.2135 | (0.0813; 0.5612) |
| 25°C | 20°C | 0.5068 | (0.2094; 1.2268) |
| 30/15°C | 20°C | 0.3339 | (0.1427; 0.7812) |
| 30/15°C | 25°C | 0.6588 | (0.3157; 1.3751) |
| Solution |  |  |  |
| pure water | GA3 | 0.8676 | (0.4744; 1.5866) |

*Odds ratio for level A relative to level B*

**Model Summary**

| **Deviance R-Sq** | **Deviance R-Sq(adj)** | **AIC** | **AICc** | **BIC** |
| --- | --- | --- | --- | --- |
| 86.33% | 62.40% | 56.50 | 62.50 | 60.36 |

**Analysis of Variance**

|  |  | **Wald Test** | |
| --- | --- | --- | --- |
| **Source** | **DF** | **Chi-Square** | **P-Value** |
| Regression | 4 | 13.08 | 0.011 |
| Temperature | 3 | 12.90 | 0.005 |
| Solution | 1 | 0.21 | 0.645 |


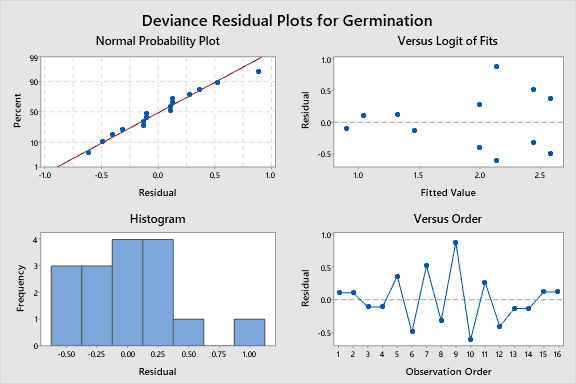

Supplement: Supplemental Information 1 [file peerj-12-16839-s001.docx]
